# Microbiome Aggregated Traits and Assembly Are More Sensitive to Soil Management than Diversity

**DOI:** 10.1128/mSystems.01056-20

**Published:** 2021-05-27

**Authors:** Andrew L. Neal, David Hughes, Ian M. Clark, Janet K. Jansson, Penny R. Hirsch

**Affiliations:** a Department of Sustainable Agriculture Science, Rothamsted Research, North Wyke, Devon, United Kingdom; b Department of Computational and Analytical Sciences, Rothamsted Research, Harpenden, Hertfordshire, United Kingdom; c Department of Sustainable Agriculture Science, Rothamsted Research, Harpenden, Hertfordshire, United Kingdom; d Biological Sciences Division, Pacific Northwest National Laboratory, Richland, Washington, USA; University of California San Diego

**Keywords:** agriculture, community-aggregated traits, diversity, microbiome, soil, species neutral assembly, tillage

## Abstract

How soil is managed, particularly for agriculture, exerts stresses upon soil microbiomes, resulting in altered community structures and functional states. Understanding how soil microbiomes respond to combined stresses is important for predicting system performance under different land use scenarios, aids in identification of the most environmentally benign managements, and provides insight into how system function can be recovered in degraded soils. We use a long-established field experiment to study the effects of combined chronic (press) disturbance of the magnitude of organic carbon inputs with acute (pulse) effects of physical disturbance by tillage and chemical disturbance due to inorganic fertilization and pesticide application. We show that because of the variety of ways it can be assessed, biodiversity—here based on microbial small subunit rRNA gene phylotypes—does not provide a consistent view of community change. In contrast, aggregated traits associated with soil microbiomes indicate general loss of function, measured as a reduction of average genome lengths, associated with chronic reduction of organic inputs in arable or bare fallow soils and altered growth strategies associated with rRNA operon copy number in prokaryotes, as well as a switch to pathogenicity in fungal communities. In addition, pulse disturbance by soil tillage is associated with an increased influence of stochastic processes upon prokaryote community assembly, but fungicide used in arable soils results in niche assembly of fungal communities compared to untilled grassland. Overall, bacteria, archaea, and fungi do not share a common response to land management change, and estimates of biodiversity do not capture important facets of community adaptation to stresses adequately.

**IMPORTANCE** Changes in soil microbiome diversity and function brought about by land management are predicted to influence a range of environmental services provided by soil, including provision of food and clean water. However, opportunities to compare the long-term effects of combinations of stresses imposed by different management approaches are limited. We exploit a globally unique 50-year field experiment, demonstrating that soil management practices alter microbiome diversity, community traits, and assembly. Grassland soil microbiomes are dominated by fewer—but phylogenetically more diverse—prokaryote phylotypes which sustain larger genomes than microbiomes in arable or bare fallow soil maintained free of plants. Dominant fungi in grassland soils are less phylogenetically diverse than those in arable or fallow soils. Soil tillage increases stochastic processes in microbiome assembly: this, combined with reduced plant biomass, presents opportunities for organisms with a capacity for pathogenesis to become established in stressed soils.

## INTRODUCTION

One consequence of the biodiversity of microorganisms in soils (1, 2) is that historically responses of below-ground communities to environmental or land use change were thought to be largely inconsequential to ecosystem processes (3). This stemmed from an assumption that although functional diversity in soils can be high, it is typically exceeded by the number of extant soil microbial species. It is generally assumed from this richness of species that soil biological systems have high levels of functional redundancy. However, soil microbial community composition and function have been shown to be sensitive to land use and climatic change, including CO_2_ increases, inorganic fertilization, temperature changes, and carbon amendments (4). Recovery of community function to predisturbance states is typically limited, particularly by long-term (chronic) disturbances (5). Understanding the effects of land management upon soil microbial diversity is important because soil microbes are responsible for the provision of a significant number of environmental services (6, 7). While the previous two decades have seen an increase in our understanding of the effects of individual physical or chemical disturbances upon microbial populations in soil, there is still limited information relating to the more realistic combined effects of physical and chemical or press and pulse disturbances (5). Arguably, the greatest disturbances to soil and associated microbial communities result from agricultural practices.

Agricultural management is associated with losses of soil organic carbon (8); harvesting limits the input of plant material, typically to just roots and stubble in arable systems, and tillage accelerates microbial decomposition of soil organic matter. Associated mechanical activity also induces soil compaction. Comparison of soils from permanently untilled grassland and arable field experiments (9) indicate that grassland soils show greater physical stability (to compression and wet/dry cycles) and biological functional stability (to temperature and metal toxicity). The loss of stability in arable soils is largely related to management effects on soil organic carbon (9).

Identifying any effects of disturbance arising from agricultural practice upon the phylogenetic assemblage and diversity of soil microbial communities is not trivial. Carbon turnover in soil typically occurs over decennial temporal scales (10). Studies of the effects of persistent soil management must account for such long temporal scales if they are to assess maximal changes in communities (5). This limits the practicality of laboratory-based experiments, but controlled field manipulations lasting many decades provide opportunities to investigate community responses to the combination of disturbances brought about by altered land management. One example of such field-scale manipulation is the Rothamsted Highfield Ley-Arable experiment, set on soil that has been under permanent grass since at least 1838. The experiment compares original grassland with continuous arable management (established in 1948) as well as bare fallowed soil, kept free of vegetation and other organic inputs (established in 1959) in the same soil and exposed to identical climatic conditions. Over this period, bare fallowed soils have become depleted in labile organic carbon and enriched in persistent organic carbon (11), and total organic carbon has been reduced to a greater extent than in arable soil. There has also been observable progressive shifts, from grassland to arable and bare fallow, in the distribution of organic carbon between different pools in the three soil managements, particularly a relative decline in discrete organic particles independent of stable soil aggregates, and a corresponding increase in the proportion of organic particles encapsulated in stable aggregates (12). Confirmation of this apparent shift in soil structure has been provided by high-resolution X-ray computed tomography (13). This long-established field experiment presents a unique opportunity to study the combined effects of press disturbance of the magnitude of organic carbon inputs (78 Mg ha^−1^ annum^−1^ from perennial grass and forbs to grassland soils, 46 Mg ha^−1^ annum^−1^ derived from annual wheat crops to arable soils, and none in bare fallow soils [14]) with pulse effects of physical disturbance by tillage (once a year in arable soils, three or four times a year in bare fallowed soils, and never in grassland soils) upon microbial communities: contemporaneous grassland effectively represents the predisturbance state of arable and bare fallow soils, but also accounting for time as a covariate.

Many studies of soil microbial diversity are limited by their reliance upon the sequencing of amplicons of small subunit rRNA (SSU rRNA) genes which do not capture the full environmental diversity (15, 16). We generated shotgun metagenome data sets from DNA extracted directly from soils subject to the three land managements, thus avoiding biases often encountered in amplicon-based diversity estimation (17, 18). Metagenome reads with homology to prokaryotic or fungal SSU rRNA genes were not clustered but analyzed individually using an evolutionary placement algorithm. This approach increases the accuracy of taxonomic identification and considers a more complete range of biodiversity represented in sequenced organisms. Microbial studies also routinely employ Shannon entropy and the Simpson index as measures of diversity; however, both are sensitive to the numbers of low-abundance organisms which is well established to be associated with sampling effort. The measures are not based upon the same units—Shannon entropy has units of information, while Simpson’s index is a probability—making direct comparison meaningless. Finally, neither behave in an intuitive linear fashion, even when relative abundances are equal (19). To avoid these issues, we described diversity using Hill numbers, with units of effective number of phylotypes; phylotype richness, Shannon diversity (the exponential of Shannon entropy), and Simpson diversity (the inverse of Simpson concentration) (19). We used shotgun metagenomes generated directly from DNA extracted from soils subject to the contrasting regimes in combination with these meaningful measures of diversity to re-examine three established hypotheses relating to the structure and phylogenetic diversity of soil prokaryotic and fungal communities. The first hypothesis is that reduced opportunity space (including reduced bioavailability of nutrients) resulting from arable and bare fallow managements will be reflected in reduced diversity of microbial communities compared to communities associated with grassland. The second hypothesis is that the reduced opportunity space, particularly as it relates to the diversity of organic matter inputs, will also be reflected in reduced average genome lengths observed in prokaryotes associated with arable and bare fallow soils and environment-associated shifts in 16S rRNA gene copy number. The third hypothesis is that physical disturbance associated with arable and bare fallow managements will result in greater heterogeneity of community assemblages (i.e., β-diversity) between individual plots due to the influence of stochastic processes upon community assembly.

## RESULTS

### Community-aggregated traits.

**FIG 1:**
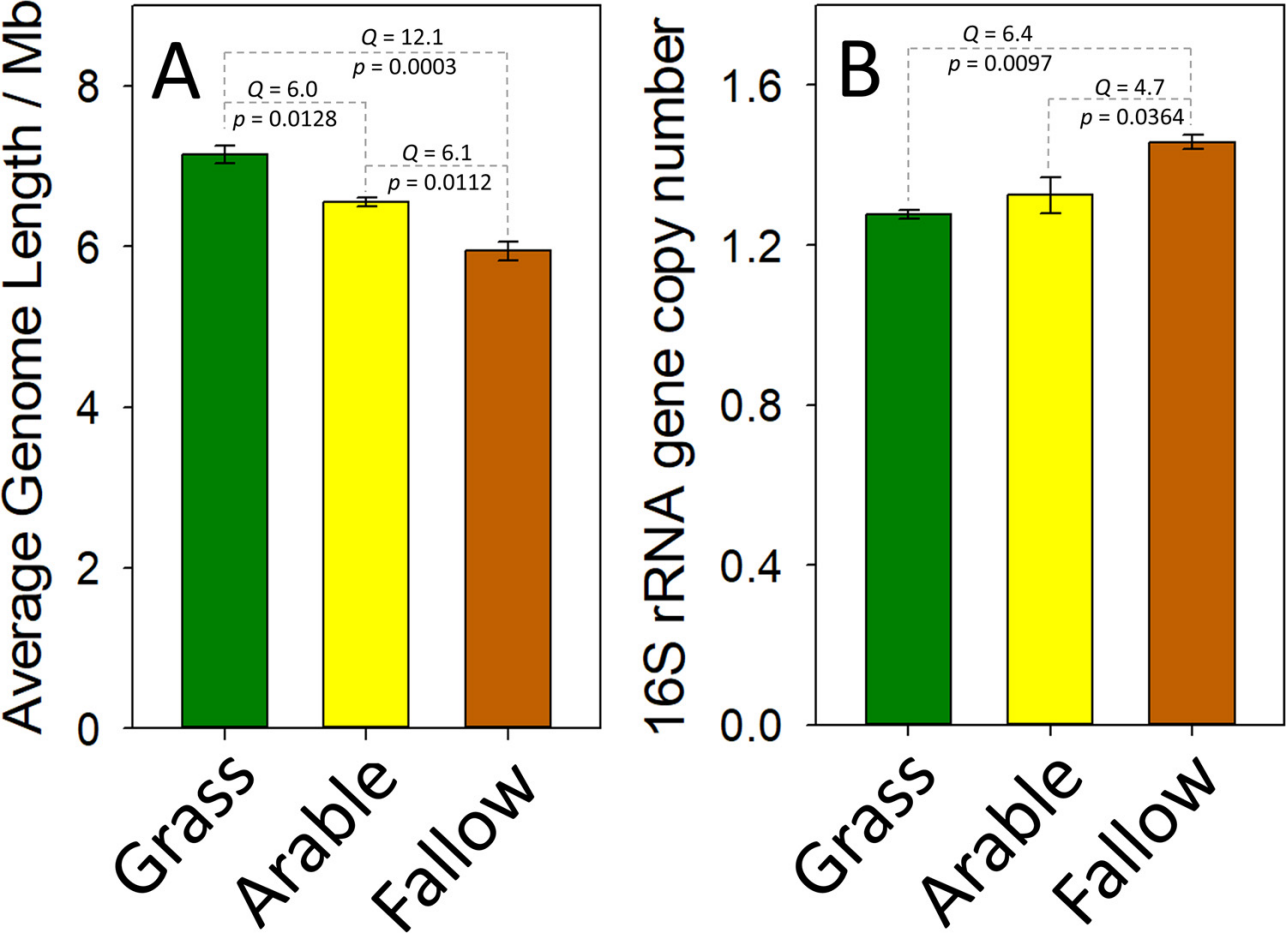
Aggregated traits of microbiomes associated with soil of the Highfield Ley-Arable field experiment. The average genome length (A) and 16S rRNA gene copy number (B) were determined from shotgun metagenomes generated from grassland (green), arable (yellow), and bare fallow (brown) soils. In each case, the mean value ± standard error of the mean (error bar) are shown. Comparisons associated with significant trait differences are indicated by dashed lines, and the associated Tukey-Kramer Studentized *Q* and probability (*p*) are given.

There was a significant difference in average genome lengths (AGL) associated with metagenomes from each land management (analysis of variance [ANOVA], *F*_2,6_ = 36.7, *P* = 0.0004, ω^2^ = 0.888). AGL was 596.3 kb and 1.204 Mb larger in grassland soil than in arable or bare fallow soils, respectively (Fig. 1A). Significant differences between land managements were also observed for 16S rRNA gene average copy number (ACN) (ANOVA, *F*_2,6_ = 10.9, *P* = 0.0100, ω^2^ = 0.688). ACN was significantly greater in bare fallow soil than in either arable or grassland soils (Fig. 1B).

### SSU rRNA gene phylogenetic placement.

Phylotype abundance is provided in Data Set S1 in the supplemental material. Bacteria in soils associated with the three land managements were dominated by *Acidobacteria*, including Luteitalea pratensis (*Vicinamibacteraceae*, *Acidobacteria* subdivision 6), “*Candidatus* Solibacter usitatus” (*Ca*. Solibacter usitatus) (*Solibacteraceae*, *Acidobacteria* subdivision 3), *Chloracidobacterium thermophilum* (*Chloracidobacterium*, *Acidobacteria* subdivision 4), the *Gemmatimonadete Gemmatirosa kalamazoonesis*, and the *Verrucomicrobium Ca*. Xiphinematobacter sp. (see Fig. S1 in the supplemental material). A second, less numerous cluster of phylogenetic placements was associated with organisms of the *Terrabacteria* group, including Fimbriimonas ginsengisoli (*Armatimonadetes*) and *Thermobaculum terrenum* (unclassified *Terrabacteria* group) among others. The most abundant *Proteobacteria* were *Rhodoplanes* sp. strain Z2-YC6860 (*Rhizobiales*) and Sphingomonas ginsengisoli (*Sphingomonadales*), both alphaproteobacteria and the unclassified betaproteobacterium GR16-43. Archaea were dominated by *Ca*. Korarchaeum cryptofilum and the closely related *Ca*. Prometheoarchaeum syntrophicum which outnumbered other placements (Fig. S2). Other abundant organisms included *Ca*. Mancarchaeum acidiphilum, the *Thermoprotei* crenarchaeotes Caldivirga maquilingensis, Pyrobaculum arsenaticum, and Sulfolobus acidocaldarius and the euryarchaeotes Methanobrevibacter ruminantium (*Methanobacteriales*), Methanopyrus kandleri (*Methanopyrales*), and Methanococcus vannielii (*Methanococcales*). There were fewer dominant taxa for fungi than for bacteria or archaea (Fig. S3). The most abundant fungus in all soils was *Conidiobolus obscurus*, a member of the Zoopagomycota. Other abundant fungi included *Brunneoclavispora bambusae* (Dothideomycetes), *Gongronella orasabula* (Mucoromycetes), *Cornuvesica acuminata* (Sordariomycetes), and *Yarrowia osloensis* (Saccharomycetes).

10.1128/mSystems.01056-20.1 FIG S1 Phylogenetic comparison of bacterial 16S rRNA phylotype assemblages in grassland (green), arable (yellow), and bare fallow (brown) soils of the Highfield Ley-Arable field experiment. The most abundant organisms are identified on branch tips of each maximum likelihood SSU rRNA gene phylogram. The placement and symbol size are scaled to reflect relative abundance across the nine samples. Replicates for each land management are represented by different placement shapes (circles, squares, or triangles). Download FIG S1, TIF file, 0.2 MB. Copyright © 2021 Neal et al. 2021 Neal et al. https://creativecommons.org/licenses/by/4.0/ This content is distributed under the terms of the Creative Commons Attribution 4.0 International license.

10.1128/mSystems.01056-20.2 FIG S2 Phylogenetic comparison of archaeal 16S rRNA phylotype assemblages in grassland (green), arable (yellow), and bare fallow (brown) soils of the Highfield Ley-Arable field experiment. The most abundant organisms are identified on branch tips of each maximum likelihood SSU rRNA gene phylogram. The placement and symbol size are scaled to reflect relative abundance across the nine samples. Replicates for each land management are represented by different placement shapes (circles, squares, or triangles). Download FIG S2, TIF file, 0.1 MB. Copyright © 2021 Neal et al. 2021 Neal et al. https://creativecommons.org/licenses/by/4.0/ This content is distributed under the terms of the Creative Commons Attribution 4.0 International license.

10.1128/mSystems.01056-20.3 FIG S3 Phylogenetic comparison of fungal 18S rRNA phylotype assemblages in grassland (green), arable (yellow), and bare fallow (brown) soils of the Highfield Ley-Arable field experiment. The most abundant organisms are identified on branch tips of each maximum likelihood SSU rRNA gene phylogram. The placement and symbol size are scaled to reflect relative abundance across the nine samples. Replicates for each land management are represented by different placement shapes (circles, squares, or triangles). Download FIG S3, TIF file, 0.1 MB. Copyright © 2021 Neal et al. 2021 Neal et al. https://creativecommons.org/licenses/by/4.0/ This content is distributed under the terms of the Creative Commons Attribution 4.0 International license.

10.1128/mSystems.01056-20.8 DATA SET S1 Taxonomy and abundance of bacterial, archaeal, and fungal small subunit ribosomal RNA gene phylotypes identified in metagenomes generated from grassland, arable, and bare fallow treatment soils of the Highfield Ley-Arable experiment. Taxonomy was inferred using a phylogenetic placement algorithm. Phylotypes associated with internal nodes of each phylogram are identified by the | symbol, which indicates a placement midway between phylogram branch tips identified on either side of the symbol. Download Data Set S1, XLSX file, 0.9 MB. Copyright © 2021 Neal et al. 2021 Neal et al. https://creativecommons.org/licenses/by/4.0/ This content is distributed under the terms of the Creative Commons Attribution 4.0 International license.

### Abundance-sensitive measures of SSU rRNA sequence diversity.

Estimates of sample coverage (*C*) for each gene were not significantly different across the land managements (Fig. S4), indicating that direct sample comparison was reasonable. The three marker genes present in the soils were not censused equally. For the bacterial 16S rRNA gene, *C* ranged from 97.0 to 98.5%. This was less than estimates for the archaeal 16S rRNA gene (*C *= 99.8 to 99.9%), but greater than estimates for the fungal 18S rRNA gene (*C *= 94.4 to 97.1%). This probably reflects a greater abundance of prokaryote than fungal SSU rRNA phylotypes and indicates that greater sequencing effort is required to capture the complete biodiversity, particularly of fungi.

10.1128/mSystems.01056-20.4 FIG S4 Sample size-based interpolation (solid line) and extrapolation (dashed line) of SSU rRNA gene phylotype coverage (C). Data points represent the observed coverage and number of phylotypes for each data set. Shaded areas represent the 95% confidence intervals of the coverage estimates. Data for bacterial and archaeal 16S rRNA genes and the fungal 18S rRNA gene are shown for grassland (green), arable (yellow), and bare fallow (brown) soils of the Highfield Ley-Arable field experiment. Download FIG S4, TIF file, 0.2 MB. Copyright © 2021 Neal et al. 2021 Neal et al. https://creativecommons.org/licenses/by/4.0/ This content is distributed under the terms of the Creative Commons Attribution 4.0 International license.

**FIG 2:**
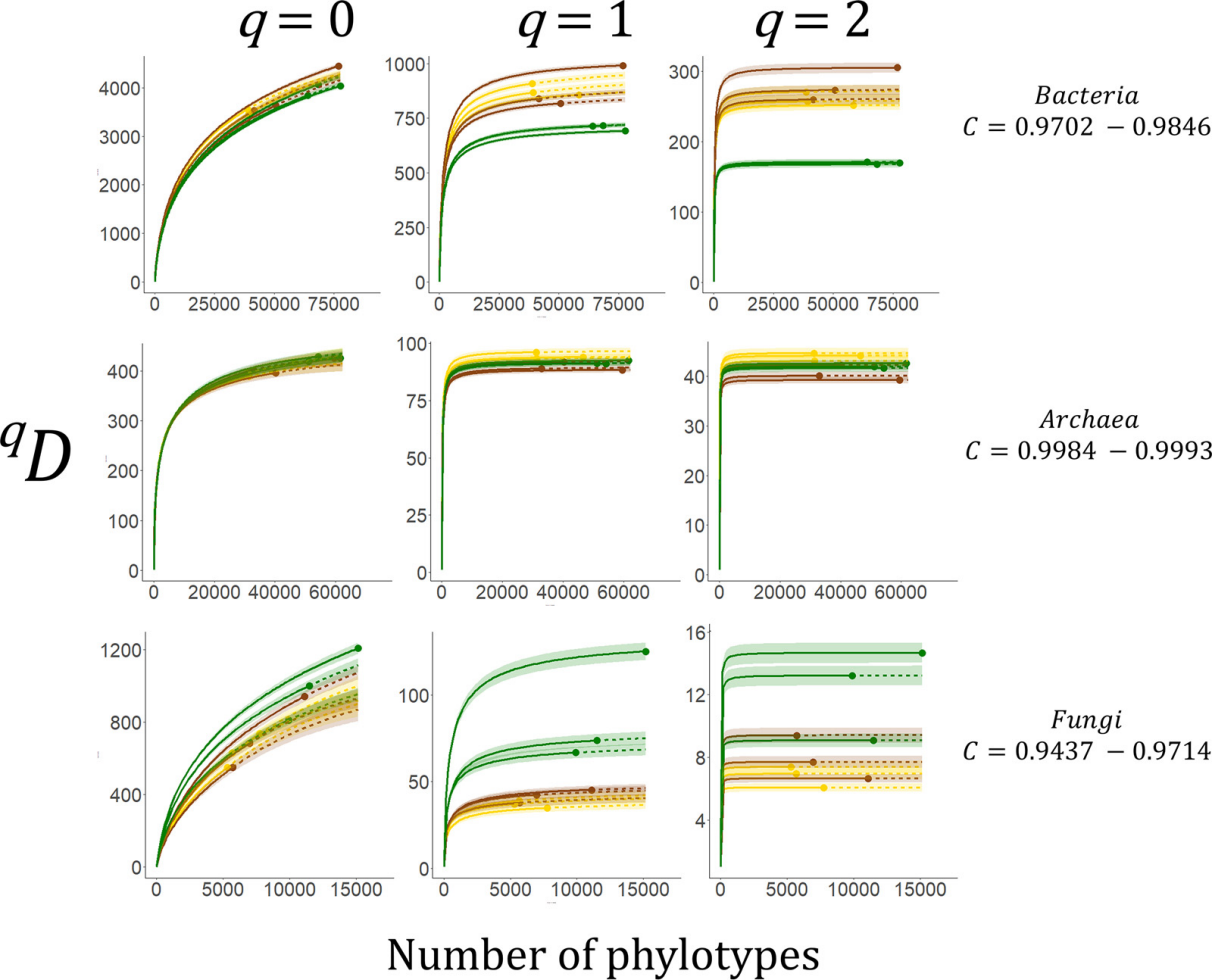
Sample size-based interpolation (solid line) and extrapolation (dashed line) of SSU rRNA gene phylotype diversity (*D*) of order *q*, *^q^D: q *= 0 (phylotype richness, left panel), *q *= 1 (Shannon diversity, middle panel), and *q *= 2 (Simpson diversity, right panel). Data points represent the observed *^q^D* and number of phylotypes for each data set. Shaded areas represent the 95% confidence intervals of the diversity estimates. Diversity is presented as the effective number of phylotypes. Data for bacterial and archaeal 16S rRNA gene and the fungal 18S rRNA gene are shown for grassland (green), arable (yellow), and bare fallow (brown) soils of the Highfield Ley-Arable field experiment. The observed range in sample coverage (*C*) for each gene is given. Individual sample coverages are shown in Fig. S1 in the supplemental material.

To test the hypothesis that reduced niche space in arable and bare fallow soils is reflected in reduced microbial diversity compared to grassland, we examined abundance-sensitive sequence diversity for each marker gene. Individual- (Fig. 2) and sample coverage-based (Fig. S5) estimates of phylotype richness (^0^*D*) indicated considerable overlap in estimate 95% confidence intervals and no consistent effect of treatment. This was particularly evident for prokaryotic 16S rRNA genes. There were no significant effects of land management upon ^0^*D* for any marker gene (largest ω^2^ = 0.383, fungal 18S rRNA gene, ANOVA *F*_2,6_ = 3.8, *P* = 0.086). Differences between land managements were more evident for ^1^*D* (Shannon diversity, weighting phylotypes in proportion to their frequency and thus representing the diversity of “common” phylotypes) and ^2^*D* (Simpson diversity, placing more emphasis on the frequencies of abundant phylotypes while discounting rare phylotypes, representing the diversity of “dominant” phylotypes). These differences in diversity were kingdom dependent. There was a significant land management effect upon ^1^*D* associated with the bacterial 16S rRNA gene (ANOVA *F*_2,6_ = 9.1, *P* = 0.015, ω^2^ = 0.642). Grassland was associated with significantly lower ^1^*D* than soils from the other managements (smallest difference, grassland versus arable, Tukey-Kramer Studentized *Q* = 5.1, *P* = 0.025). There was a more pronounced management effect on ^2^*D* (ANOVA *F*_2,6_ = 48.1, *P* < 0.001, ω^2^ = 0.913), grassland again being associated with significantly lower diversity than the other soils (smallest difference, grassland versus arable *Q *= 10.7, *P* < 0.001) which were equally diverse. Diversity of the archaeal 16S rRNA gene was also influenced significantly by management (ANOVA ^1^*D* − *F*_2,6_ = 8.3, *P* = 0.019, ω^2^ = 0.619; ^2^*D* − *F*_2,6_ = 8.2, *P* = 0.019, ω^2^ = 0.615). For both measures, arable soils were significantly more diverse than bare fallow soils (smallest difference ^2^*D*, *Q *= 5.7, *P* = 0.016), but there was no significant difference between grassland and arable soil diversities. For the fungal 18S rRNA gene, a significant influence of land management was again apparent (ANOVA ^1^*D* − *F*_2,6_ = 7.0, *P* = 0.027, ω^2^ = 0.573; ^2^*D* − *F*_2,6_ = 7.1, *P* = 0.026, ω^2^ = 0.575). For ^1^*D*, grassland was significantly more diverse than either arable or bare fallow soils (smallest difference, grassland versus bare fallow *Q *= 4.4, *P* = 0.049); however, in the case of ^2^*D*, only the difference between grassland and arable soils was significant (*Q *= 5.0, *P* = 0.028). The trends indicated that grassland soils were associated with significantly lower diversity of common (^1^*D*) and dominant (^2^*D*) bacterial phylotypes. This was reversed for fungi, where grassland was associated with the highest ^1^*D* and ^2^*D* phylotype diversities. There was also considerable variation between grassland replicates. For these genes, diversity in arable and bare fallow soils was similar. Archaeal sequence abundance distributions were markedly different from those observed for bacteria and fungi in the sense that the greatest sequence diversities were observed in soils managed as arable. Analysis of abundance-sensitive phylotype diversity provides insight into abundance distributions associated with soils from the different treatments. No phylogenetic information is considered, even though it is inherent in the sequences upon which the analysis is based.

10.1128/mSystems.01056-20.5 FIG S5 Sample coverage-based interpolation (solid line) and extrapolation (dashed line) of SSU rRNA gene phylotype diversity (*D*) of order *q*, *^q^D: q *= 0 (species richness, left panel), *q *= 1 (Shannon diversity, middle panel), and *q * = 2 (Simpson diversity, right panel). Data points represent the observed *^q^D* and coverage for each data set. Shaded areas represent the 95% confidence intervals of the diversity estimates. Diversity is presented as the effective number of species. Data for bacterial and archaeal 16S rRNA genes and the fungal 18S rRNA gene are shown for grassland (green), arable (yellow), and bare fallow (brown) soils of the Highfield Ley-Arable field experiment. Download FIG S5, TIF file, 0.2 MB. Copyright © 2021 Neal et al. 2021 Neal et al. https://creativecommons.org/licenses/by/4.0/ This content is distributed under the terms of the Creative Commons Attribution 4.0 International license.

### Phylogeny-sensitive measures of SSU rRNA sequence diversity.

As an additional test, we calculated sequence phylogenetic diversity (PD) using a one-parameter family of α-diversity measures—balance-weighted phylogenetic diversity (*BWPD*_θ_)—based upon phylogenetic placement of metagenome reads on each reference marker gene phylogram. Profiles show the phylogenetic diversity of increasingly more abundant organisms, akin to *^q^D* described above: *BWPD*_0_ takes no account of phylotype abundance, while *BWPD*_1_ considers the most abundant phylotypes. Resulting profiles are shown in Fig. S6. They demonstrate a common, highly uneven phylogenetic diversity-abundance distribution but with observable differences between land uses.

10.1128/mSystems.01056-20.6 FIG S6 Comparison of phylogenetic diversity profiles of SSU rRNA gene phylotype assemblages associated with grassland (green), arable (yellow), and bare fallow (brown) soils of the Highfield Ley-Arable field experiment based upon a one-parameter family of diversity measures, *BWPD*_θ_, that interpolates between classical phylogenetic diversity (PD, *θ* = 0) and an abundance-weighted extension of PD (*θ* = 1). Download FIG S6, TIF file, 0.1 MB. Copyright © 2021 Neal et al. 2021 Neal et al. https://creativecommons.org/licenses/by/4.0/ This content is distributed under the terms of the Creative Commons Attribution 4.0 International license.

**FIG 3:**
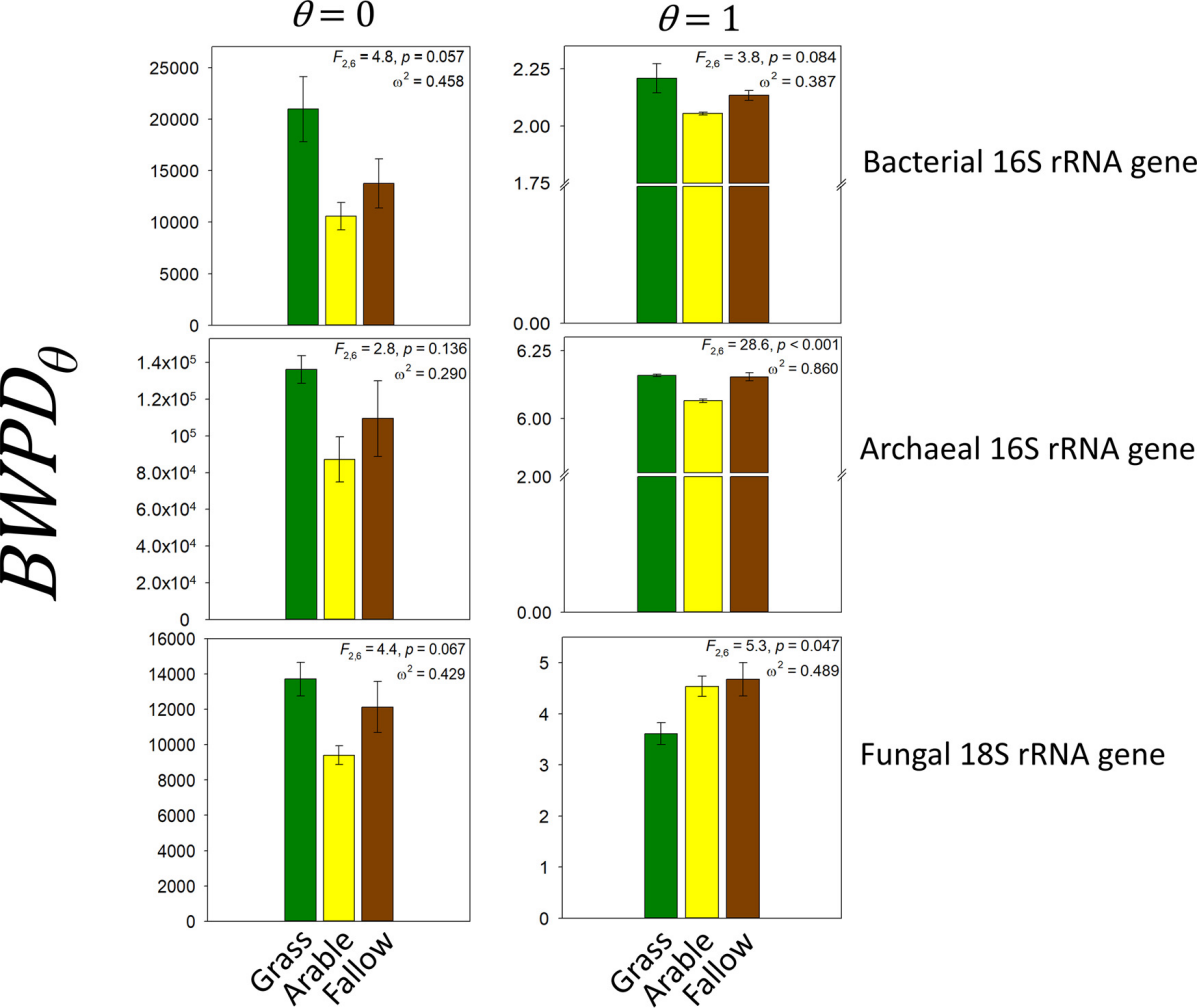
Comparison of phylogenetic diversity of SSU rRNA gene phylotype assemblages associated with grassland (green), arable (yellow), and bare fallow (brown) soils of the Highfield Ley-Arable field experiment based upon a one-parameter family of diversity measures, *BWPD*_θ_, that interpolates between classical phylogenetic diversity (PD, *θ* = 0, left panel) and an abundance-weighted extension of PD (*θ* = 1, right panel). The mean ± standard error of the mean *BWPD*_θ_ together with results of a one-factor analysis of variance and observed effect size (ω^2^) are shown for each gene. *BWPD*_θ_ profiles are shown in Fig. S6.

These differences are illustrated best by considering the extremes of PD profiles: *BWPD*_0_ (Faith’s PD, representing the sum of lengths of phylogram branches spanning all community members), and its abundance-weighted extension (*BWPD*_1_) shown in Fig. 3. As with the response of ^0^*D* above, there was no significant effect of land management upon *BWPD*_0_ associated with any biomarker gene, although a clear consistent trend of arable soils being associated with the lowest PD was evident. This observed lack of a treatment effect upon *BWPD*_0_ may reflect a remarkable resistance of soil microbiome PD to environmental change. However, alternatively, it may reflect a relative lack of statistical power of comparing three replicates per land management or using only 2 g of soil from which to extract nucleic acids which, even though soil samples were well mixed during processing, may not capture the full extent of spatial heterogeneity in the soil communities. Irrespective of this, ω^2^ estimates suggested that archaeal *BWPD*_0_ was the least sensitive to the different treatments, consistent with observations derived from *^q^D* measures of phylotype diversity. There was a significant effect of management upon archaeal 16S rRNA gene *BWPD*_1_, and ω^2^ estimates suggested that archaea were in this case the most sensitive to the imposed managements when both phylogeny and abundance were considered. *BWPD*_1_ was significantly lower in arable soil (6.05 ± 0.006, mean ± standard error) than in grassland (6.16 ± 0.004, *Q *= 9.5, *P* = 0.0013) or bare fallow (6.15 ± 0.015, *Q *= 9.0, *P* = 0.0018) soils. There was no significant difference between grassland or bare fallow soil archaeal *BWPD*_1_. There was also a significant effect of treatment upon *BWPD*_1_ associated with the fungal 18S rRNA gene. In this case, grassland soil was associated with lower *BWPD*_1_ (3.61 ± 0.213) than either arable (4.54 ± 0.197) or bare fallow (4.68 ± 0.324) soils. There was, however, no statistically significant difference between bare fallow and grassland soils (*Q *= 4.2, *P* = 0.055), having the extremes of fungal *BWPD*_1_.

### Comparison of SSU rRNA gene sequence assemblages.

**FIG 4:**
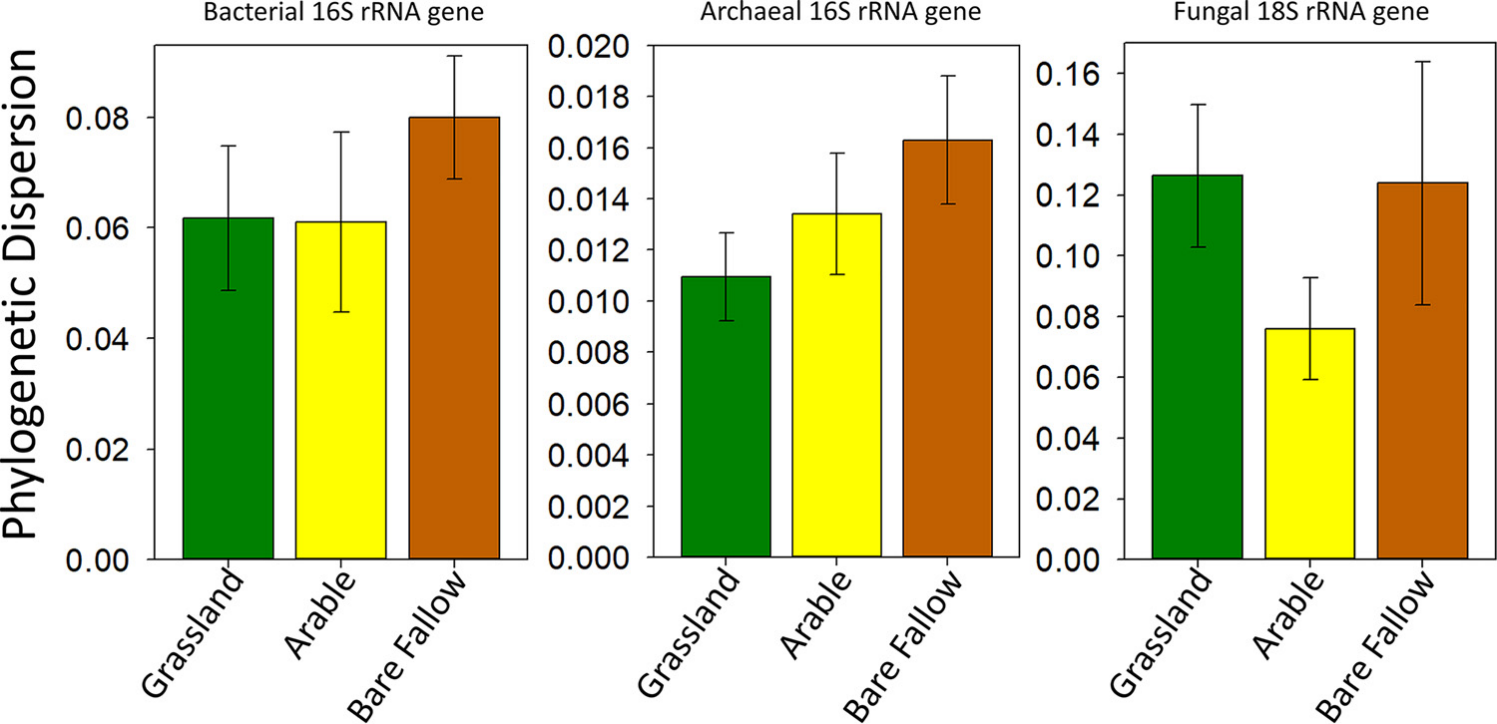
Phylogenetic dispersion associated with SSU rRNA phylotype assemblages in grassland (green), arable (yellow), and bare fallow (brown) soils of the Highfield Ley-Arable field experiment. Phylogenetic dispersion was estimated based upon the multivariate deviation of each replicate community from the centroid of each land management group in Euclidean space, based upon Kantorovich-Rubinstein phylogenetic distances between each phylotype assemblage. The mean ± 95% confidence interval (error bar) are shown for each soil.

Our third hypothesis relating to processes controlling community assembly in disturbed soils predicted that physical or chemical disturbance associated with arable and bare fallow management would result in greater assemblage heterogeneity than is observed for undisturbed grassland soils. To test this, we generated Kantorovich-Rubinstein (KR) distance metrics, based upon the distribution of homologous reads associated with each land management on reference phylograms. We calculated the multivariate KR deviation of each replicate community from each land management centroid in Euclidean space (phylogenetic dispersion). The rationale was that where a disturbance (for example, incorporation of fungicide as a seed coat in arable soils) resulted in strong environmental filtering, phylogenetic dispersion would be lower than that for grassland soil. Where community assembly in disturbed soil was subject to a strong influence of stochastic processes, phylogenetic dispersion would be greater than in grassland soil. The observed relationships between the communities in each soil are shown in Fig. 4. In bare fallow soils, there is greater bacterial phylogenetic dispersion than is observed in grassland soils, although there is overlap of 95% confidence intervals around the means. This provides evidence of an increased influence of stochastic processes in bacterial community assembly in bare fallow soils than grassland soils. Bacterial community phylogenetic dispersion in arable soils is indistinguishable from grassland soil communities. The trend of increased community phylogenetic dispersion in disturbed soils is more evident for archaea, where phylogenetic dispersion is greater within arable and bare fallow soil communities. In this instance, the 95% confidence intervals suggest significantly greater dispersion between communities in bare fallow soil than in grassland soil. The response of fungal soil communities to disturbance is not consistent with an increased influence of stochasticity observed for prokaryotes. There was significantly less phylogenetic dispersion between fungal communities in arable soil compared to communities in grassland soil. This suggests increased environmental filtering during community assembly. Environmental filtering was not observed for fungal communities in bare fallow soils which were associated with similar phylogenetic dispersion as grassland soil communities.

A significant effect of land management upon sequence assemblages of bacterial 16S rRNA (permutational multivariate analysis of variance [PERMANOVA], pseudo*-F*_2,6_ = 16.3, probability estimation based upon 99,999 permutations [*p*_perm_] = 0.0034), archaeal 16S rRNA (PERMANOVA, pseudo*-F*_2,6_ = 8.0, *p*_perm_ = 0.0036), and fungal 18S rRNA (PERMANOVA, pseudo*-F*_2,6_ = 3.0, *p*_perm_ = 0.0105) phylotypes was detected. *Post hoc* pairwise comparisons indicated that prokaryote assemblages were significantly different between all land managements; in both cases, the smallest pseudo*-t* was associated with the arable versus bare fallow comparison (bacteria, pseudo*-t *= 3.0, Monte Carlo probabilities [*p*_MC_] = 0.0084; archaea pseudo*-t *= 0.1, *p*_MC_ = 0.0301). Land management differences were more limited for the fungal 18S rRNA gene. In this case, only the comparison of assemblages in arable and grassland soils indicated a significant difference (pseudo-*t *= 2.2, *p*_MC_ = 0.0291). Associated canonical analyses of principal coordinates are shown in Fig. S7.

10.1128/mSystems.01056-20.7 FIG S7 Discriminant analysis employing canonical analysis of principal coordinates (CAP) of SSU rRNA phylotype assemblages based upon Kantorovich-Rubinstein phylogenetic distance metrics. Phylotype assemblages associated with grassland (green), arable (yellow), and bare fallow (brown) soils of the Highfield Ley-Arable field experiment are shown. For each ordination, two CAP axes were defined, based upon maximizing a leave-one-out allocation success to *a priori* land management groups. The results of permutation tests of the significance of the canonical relationships using the trace statistic (sum of canonical eigenvalues) are shown. Download FIG S7, TIF file, 0.08 MB. Copyright © 2021 Neal et al. 2021 Neal et al. https://creativecommons.org/licenses/by/4.0/ This content is distributed under the terms of the Creative Commons Attribution 4.0 International license.

**FIG 5:**
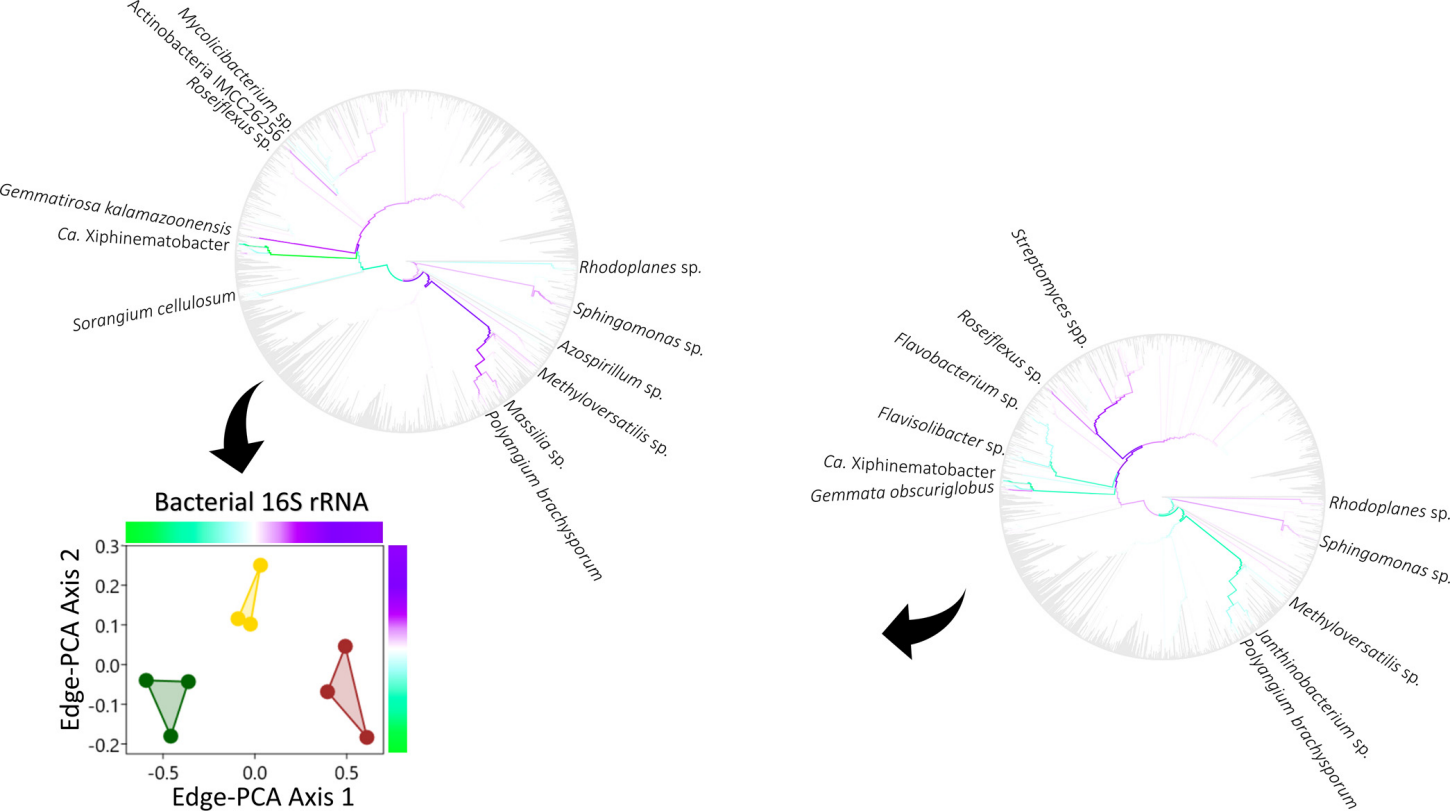
Ordination of bacterial 16S rRNA gene phylotype assemblages shown in Fig. S1, exploiting the underlying phylogenetic nucleotide sequence structure (edge-PCA). Phylotype assemblages associated with grassland (green), arable (yellow), and bare fallow (brown) soils of the Highfield Ley-Arable field experiment are separated across both edge-PCA axes. Edges associated with large eigenvectors are shown in each axis-associated color-coded phylogram, which corresponds with the axis color scales. Phylotypes associating more with grassland, arable, or bare fallow soils are identified.

**FIG 6:**
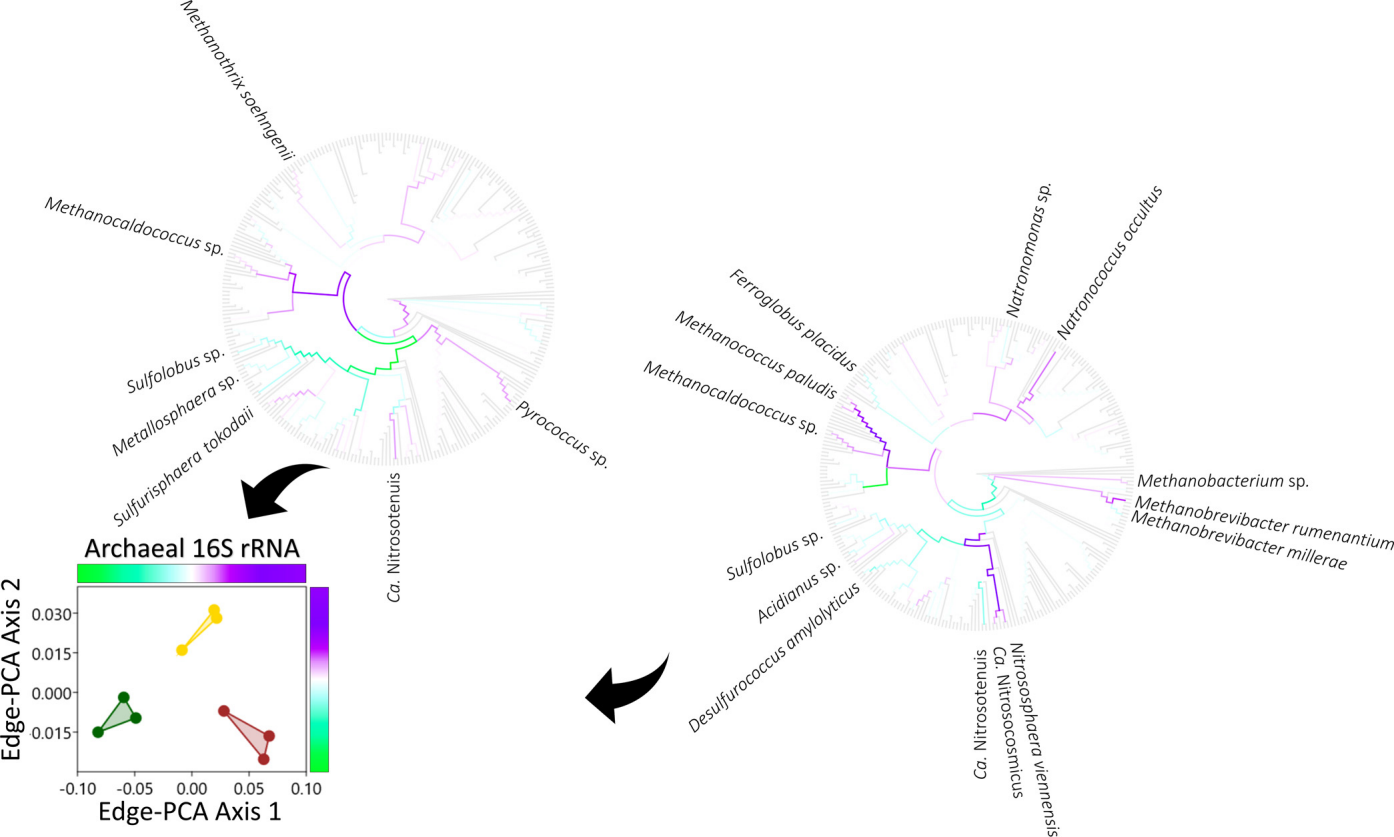
Ordination of archaeal 16S rRNA gene phylotype assemblages shown in Fig. S2, exploiting the underlying phylogenetic nucleotide sequence structure (edge-PCA). Phylotype assemblages associated with grassland (green), arable (yellow), and bare fallow (brown) soils of the Highfield Ley-Arable field experiment are separated across both edge-PCA axes. Edges associated with large eigenvectors are shown in each axis-associated color-coded phylogram, which corresponds with the axis color scales. Phylotypes associating more with grassland, arable, or bare fallow soils are identified.

To identify taxa responsible for the observed distinctiveness between land managements, we used edge-PCA (unconstrained ordination based upon principal-component analysis of the difference in placement masses across reference phylograms) to identify phylogram branches across which there was a high level of between-sample heterogeneity. Ordination of bacterial and archaeal 16S rRNA gene assemblages separated the land managements clearly in two dimensions (Fig. 5 and 6). On edge-PCA axis 1, bacteria such as *Ca.* Xiphinematobacter, *Rhodoplanes* sp., and the deltaproteobacterium Sorangium cellulosum and the crenarchaeotes *Sulfolobus* sp. and *Metallosphaera* sp. were more associated with grassland soils. The *Actinobacteria Mycolicibacterium* sp. and bacterium IMCC26256, the *Chloroflexia Roseiflexus* sp., the alphaproteobacteria *Azospirillum* sp. and *Sphingomonas* sp., the betaproteobacteria *Massilia* sp. and *Methyloversatilis* sp., the deltaproteobacterium *Polyangium brachysporum* and the *Gemmatimonadetes Gemmatirosa kalamazoonensis*, the crenarchaeote *Sulfurisphaera tokodaii*, the euryarchaeotes *Pyrococcus* sp., Methanothrix soehngenii, and *Methanocaldococcus* sp., and the thaumarchaeote *Ca*. Nitrosotenuis were all associated more with bare fallow soil. On the second axis, *Roseiflexus* sp., *Rhodoplanes* sp., *Sphingomonas* sp., the planctomycete Gemmata obscuriglobus, and the actinobacterium *Streptomyces* sp., *Methanocaldococcus* sp., and other *Methanomada* group euryarchaeotes, including *Methanococcus paludis*, *Methanobrevibacter* spp., and *Methanobacterium* sp., the halobacteria euryarchaeaotes Natronococcus occultus and *Natronomonas* sp., and the *Nitrososphaerales* thaumarchaeotes *Ca*. Nitrosocosmicus and Nitrososphaera viennensis were all more associated with arable soil.

**FIG 7:**
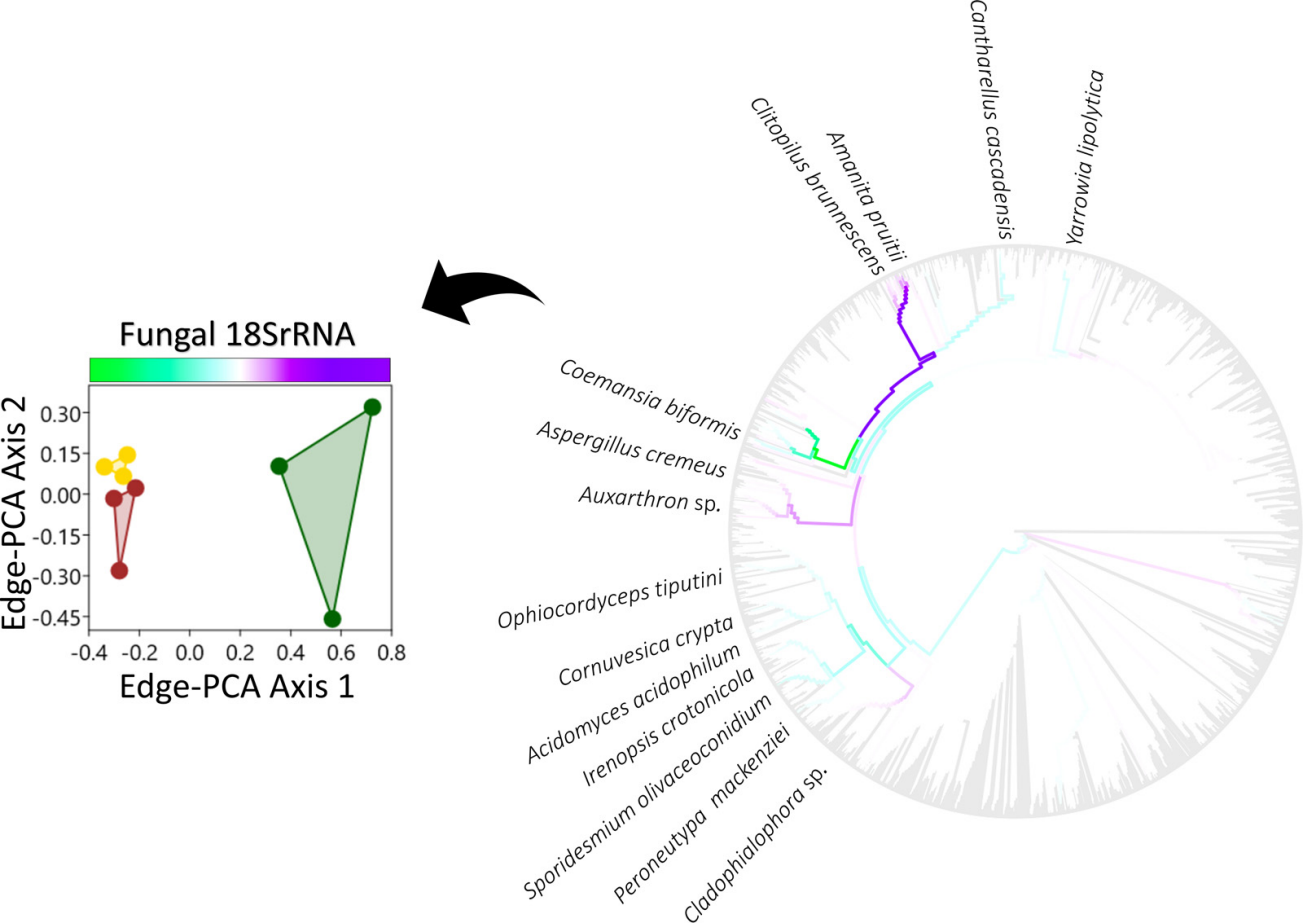
Ordination of fungal 18S rRNA gene phylotype assemblages shown in Fig. S3, exploiting the underlying phylogenetic nucleotide sequence structure (edge-PCA). Phylotype assemblages associated with grassland (green), arable (yellow), and bare fallow (brown) soils of the Highfield Ley-Arable field experiment are separated across both edge-PCA axes. Edges associated with large eigenvectors are shown in the axis-associated color-coded phylogram, which corresponds with the axis color scale. Phylotypes associating more with grassland, arable, or bare fallow soils are identified.

Edge-PCA ordination of fungal 18S rRNA gene assemblages revealed a distinctly different treatment distribution than observed for 16S rRNA genes (Fig. 7). Treatment differences were distributed only across the first axis, separating grassland assemblages from arable and bare fallow assemblages. Taxa most associated with grassland were the Agaricomycetes (Basidiomycota) *Amanita pruitii* and *Clitopilus brunnescens* and the Eurotiomycetes (Ascomycota) *Aspergillus cremeus*, *Cladophialophora* sp., and *Auxarthron* sp. Arable and bare fallow soils were most associated with the Saccharomycete (Ascomycota) Yarrowia lipolytica, the Agaricomycete *Cantharellus cascadensis*, the Kickxellomycete (Zoopagomycota) *Coemansia biformis*, the Sordariomycetes (Ascomycota) *Ophiocordyceps tiputini*, *Cornuvesica crypta*, *Sporidesmium olivaceoconidium*, *Peroneutypa mackenziei*, and *Irenopsis crotonicola*, and the Dothideomycete (Ascomycota) *Acidomyces acidophilum*. Ecological guilds associated with these taxa (Table 1) suggest grassland soil was associated more with ectomycorrhizal and saprotrophic fungi, whereas taxa more associated with arable and bare fallow soils were microfungal in growth habit and had the capacity to pathotrophy, associating with animals, plants, and lichens.

**TABLE 1:** Predictions of trophic mode, growth form, and ecological guild for the fungal species identified with shifts in community assemblages in soils^*a*^

Genus	Association	Trophic mode(s)	Growth form	Guild(s)
*Acidomyces*	Arable/fallow	Pathotroph, saprotroph, symbiotroph	Microfungus	Endophyte, plant pathogen, unknown saprotroph, wood saprotroph
*Amanita*	Grassland	Symbiotroph	Agaricoid	Ectomycorrhizal
*Aspergillus*	Grassland	Pathotroph, saprotroph, symbiotroph	Microfungus	Animal pathogen, endophyte, plant saprotroph, soil saprotroph, undefined saprotroph, wood saprotroph
*Auxarthron*	Grassland	Saprotroph	Not known	Undefined saprotroph
*Cantharellus*	Arable/fallow	Symbiotroph	Cantherelloid	Ectomycorrhizal
*Cladophialophora*	Grassland	Saprotroph	Facultative yeast	Undefined saprotroph
*Clitopilus*	Grassland	Saprotroph	Agaricoid	Undefined saprotroph
*Coemansia*	Arable/fallow	Saprotroph	Not known	Undefined saprotroph
*Cornuvesica*	Arable/fallow	Pathotroph, saprotroph	Microfungus	Plant pathogen, wood saprotroph
*Irenopsis*	Arable/fallow	Pathotroph	Not known	Plant pathogen
*Ophiocordyceps*	Arable/fallow	Pathotroph, symbiotroph	Microfungus	Animal pathogen, endophyte
*Peroneutypa*	Arable/fallow	Pathotroph	Not known	Plant pathogen
*Sporidesmium*	Arable/fallow	Pathotroph	Microfungus	Lichen parasite
*Yarrowia*	Arable/fallow	Saprotroph	Yeast	Undefined saprotroph

a Predictions are taken from FUNGuild version 1.0 (75).

## DISCUSSION

The Highfield Ley-Arable experiment soils studied here have experienced consistent management for sufficiently long periods of time for the complete extent of microbial community response to become apparent. While grassland soils effectively represent the original soil community traits, structures, and phylogeny, soils managed as arable or bare fallow continue to experience combinations of press (different levels of plant inputs) and pulse (different levels of tillage, addition of inorganic fertilizers, and wheat seed-associated pesticide combination) disturbance. Despite these long-term combinations of disturbance, the prokaryotic and fungal communities in all soils are dominated by a limited number of abundant organisms, several of which share partner-dependent lifestyles. For example, *Ca*. Xiphinematobacter sp., one of the more abundant bacteria in all metagenomes (consistently one of the 20 most abundant bacterial species [see Fig. S1 in the supplemental material]), is an obligate mutualist endosymbiont of a group of migratory plant root-ectoparasitic nematodes, Xiphinema americanum *sensu lato* (20). It has been identified in 49 of the 61 nominal species comprising the X. americanum *sensu lato* complex (21). The organism was more abundant in grassland and arable soil than in bare fallow soils (Fig. 5), and this is consistent with 16S rRNA amplicon sequencing of these microbiomes which identified a *Verrucomicrobium* as being associated with significantly different abundance between the three soils (22). Of the dominant archaeal species, two are dependent upon associations with other organisms. *Ca*. Prometheoarchaeum syntrophicum MK-D1 is a slow-growing organism that degrades amino acids syntrophically with other archaea—*Halodesulfovibrio* and *Methanogenium* in the original cocultures (23). A second organism, *Ca*. Mancarchaeum acidiphilum MIA14, lacks any genes of the central carbohydrate metabolic pathways, but degrades proteins and amino acids as part of obligate mutualistic partnerships with *Thermoplasmatales* archaea (24). The most abundant fungus in all soils was the entomopathogen *Conidiobolus obscurus*, which produces conidia that infect aphids (25, 26). Another abundant microfungus, *Cornuvesica acuminata*, requires metabolites (possibly siderophores) from other fungi for growth (27).

Compared to grasslands composed of mixed forb and grass plant species, arable and bare fallow soils provide severely limited breadths of niche space for microbes: limited diversity of plant species and reduced ranges of organic inputs. Our second hypothesis predicted that reduced opportunity space in arable and bare fallow soils would be associated with changes to prokaryotic community-aggregated traits (CATs): average genome length (AGL) and 16S rRNA gene average copy number (ACN). The effect of land management upon CATs was marked. AGL in Highfield soils ranged between 5.8 and 7.4 Mb, larger than estimates derived from marine environments (28) but consistent with data from other soils (29, 30). Within this range, prokaryotic microbiomes of arable and bare fallow soils were associated with significantly shorter AGL than grassland microbiomes (Fig. 1). Assuming an average prokaryote gene length of 0.924 kb (31), the 596.3-kb and 1.204-Mb reductions of arable and bare fallow AGL represent losses of approximately 645 and 1,300 genes per genome compared to prokaryotes in grassland soil. This suggests strong genome streamlining (32) driven by a pervasive bias toward greater numbers of nucleotide deletions than insertions in the absence of strong selective pressures to maintain genes (33). In the absence of the wide variety of organic inputs in grassland soils, a great number of genes are lost; the less diverse the inputs, the greater number of lost genes. This observation suggests that AGL within prokaryotic communities reflects the complexity of the environment, as has been suggested for individual genera (34, 35). Although we have not tested it directly here, this large reduction in AGL is likely to represent a significant reduction in functional diversity. Marked differences in microbiome potential function between these soils have been demonstrated (14). In addition, the 16S rRNA ACN suggests that microbiome responses to inputs are also altered in soils. ACN in Highfield soils ranged from 1.26 to 1.48, while in prokaryotes more generally, it is known to range between 1 and 15 (36). These low copy numbers are typical of soils and indicative of organisms in oligotrophic environments which are predicted to be under selective pressure to maintain low numbers of rRNA-encoding genes (37). ACN was statistically greater in bare fallow soil than in either arable or grassland soils, suggesting a possible shift in ecological strategy. Bacteria with greater numbers of rRNA operons show more rapid responses to substrate inputs in the laboratory (38); however, we have no evidence that the communities in bare fallow soils respond in a similar fashion. Together, these CATs suggest that microbiomes in arable and bare fallow soils have lost a significant number of genes (and associated functions) but maintain a greater number of rRNA operons, enabling a more rapid response to organic inputs when they occur. Comparing ω^2^ between CATs indicates that AGL is more sensitive to stressors than 16S rRNA gene copy number is.

Our first hypothesis predicted that reduced niche space would be reflected in lower diversity of prokaryote and fungal communities typifying each disturbed soil. We generated abundance- and phylogeny-sensitive diversity measures that suggest a nuanced response of biodiversity to land management. Abundance-insensitive measures indicated no statistically significant differences in phylotype richness (^0^*D*, Fig. 2) or phylogenetic diversity (*BWPD*_0_, Fig. 3). Lack of any statistically significant effect of land management upon ^0^*D* or *BWPD*_0_ could be a result of the low statistical power of the Highfield experiment; however, richness and PD cannot be estimated in a robust fashion (39), and our results may reflect this. However, our observations are consistent with comparisons of bacterial communities in tilled agricultural soils and untilled grassland of other long-term managed soils of the W.K. Kellogg Biological Station research site (40) and between tilled and nontilled arable soils of the Swiss Farming Systems and Tillage Experiment (41). Our observation that fungal phylotype richness was also not sensitive to tillage in arable and bare fallow soils does not support the observation of the long‐term Swiss experiment, where a distinct reduction in fungal richness has been observed in response to tillage (41). Any perceived influence of tillage upon fungal species richness based upon sequencing of amplified regions of the 18S rRNA gene (41) is likely to be subject to bias in primer amplification (42); an apparent effect of tillage may reflect a shift in fungal assemblages rather than a loss of richness. There was a consistent though not statistically significant trend associated with *BWPD*_0_—“feature diversity” (43)—where arable soil was associated with the lowest, and grassland with the highest *BWPD*_0_ for each SSU rRNA gene. It is worth noting that *BWPD*_0_ associated with arable soil was consistently lower than even that associated with bare fallow soil.

Phylotype abundance-sensitive ^1^*D*, ^2^*D*, and *BWPD*_1_ are all estimated with greater certainty, and these parameters indicated significant land management effects upon diversity. Grassland soils were associated with significantly fewer common and dominant bacterial phylotypes, suggesting a more uneven community profile. However, *BWPD*_1_ (Fig. 3) suggests that the fewer dominant phylotypes were associated with greater PD than the dominant phylotypes in arable or bare fallow soils, though not significantly greater. For the fungal 18S rRNA gene, this distribution was reversed: grassland soils were associated with a greater number of common and dominant phylotypes (Fig. 2), but these dominant phylotypes were significantly less phylogenetically diverse than dominant phylotypes in disturbed soils (Fig. 3). The greatest number of archaeal 16S rRNA gene phylotypes was observed in arable soils (Fig. 2). These were associated with significantly lower *BWPD*_1_ than either grassland or bare fallow soils (Fig. 3). Prokaryotic communities appeared to have a common phylogeny-sensitive response to land management. This assessment provides several salient observations: combinations of pulse and press disturbance in soil systems do not result in consistently reduced measures of diversity: abundance- and phylogeny-sensitive measures of diversity are necessary to generate a complete view of soil microbiome responses to disturbance, and community responses are kingdom specific.

Bacterial, archaeal, and fungal assemblages were each sensitive to management (Fig. S7) consistent with many similar studies (5). Detailed analysis of the assemblages associated with grassland, arable, and bare fallow soils studied here suggests that shifts in community structure typically do not involve dominant phylotypes. Few phylotypes associated with large edge-PCA eigenvalues in Fig. 5 to 7 were dominant as indicated in Fig. S1 to S3. Exceptions to this observation were the nematode endosymbiont *Ca*. Xiphinematobacter sp. which was more numerous in grassland than bare fallow soils, and Gemmatirosa kalamazoonesis, a representative of a group of extremely abundant soil bacteria (*Gemmatimonadetes*) well adapted to arid conditions (44) which was more numerous in bare fallow soil than grassland, consistent with previous 16S rRNA amplicon sequencing of these soils (12). A second organism most numerous in bare fallow soils was *Methyloversatilis* sp. which grows on single-carbon compounds (45), suggesting that organisms adapted to arid conditions or capable of utilizing simple carbon substrates were typical of bacteria in bare fallowed soils. Arable soils were associated with significantly higher ^1^*D* and ^2^*D*, and greater numbers of *Methanomada* and halobacteria euryarchaeotes as well as of ammonia-oxidizing Nitrososphaera viennensis and *Ca*. Nitrosocosmicus sp. These latter organisms suggest that the increased abundance-sensitive phylotype diversity but decreased phylogenetic diversity of archaea resulting from arable management may reflect regular nitrogen fertilization of these soils and is consistent with the strong association between soil nitrate and ammonia-oxidizing archaea (46). Thus, despite the pulse disturbances of tillage, fertilization, and pesticide application being confounded in the arable soil, it is possible to associate the observed shifts in abundance- and phylogeny-sensitive diversity with different disturbances. The response of fungi to land management was distinct from that of prokaryotes, since the difference in communities was expressed on only one edge-PCA dimension (Fig. 7, also Fig. S7) separating grassland from the disturbed soils and suggesting that tillage elicited this response, since bare fallow soil was neither fertilized nor received pesticide. Tillage therefore reduces the abundance-sensitive phylotype diversity of fungi but increases the phylogenetic diversity of those dominant phylotypes. Ectomycorrhizal *Amanita pruitii* and saprotrophic *Clitopilus brunnescens* were less numerous in disturbed soils than grassland. Most fungal species identified as more numerous in arable and bare fallow soils had microfungal or yeast-like growth forms (Table 1), possibly because of the effect of physical disturbance arising from tillage upon ectomycorrhizal fungi (47, 48). Fungal species which became more numerous in disturbed soils were predominantly pathotrophs of insects (*Ophiocordyceps tiputini*), plants (*Acidomyces acidophilum*, *Cornuvesica crypta*, *Irenopsis crotonicola*, and *Peroneutypa mackenziei*), and lichens (*Sporidesmium olivaceoconidium*). The differences in phylotype assemblages observed between the land managements reflect the predicted selection pressures within the soils and organismal traits.

Of equal interest to the effects of land management upon microbial diversity is the issue of how disturbance influences microbiome assembly, testing our third hypothesis. Our data support the proposition that physical pulse disturbance by tillage in arable and bare fallow soils results in increased prokaryotic phylogenetic dispersion than in nontilled grassland soils and that archaeal assembly is more sensitive to tillage than bacterial (Fig. 4). This is indicative of an increased role for species neutral assembly where community structures result from stochastic colonization and extinction processes and are influenced less by species traits (49, 50). Differences in bacterial community assembly have been observed between agricultural soils of the Wageningen Soil Health Experiment (51). In this case, assembly in organically fertilized soils was associated with greater stochasticity than in inorganically fertilized soils, possibly because of the variety of different organic treatments and the presence of weed plant species. Fire disturbance in Colorado soils dominated by ponderosa pine and Douglas firs also results in increased stochasticity in community assembly immediately after fires, but over time this is replaced by niche assembly (52). On Highfield, stochasticity is likely to arise as tillage disrupts community assembly once per year in arable soils but three or four times in bare fallow soils. Prokaryotic phylogenetic dispersion increases with the frequency of tillage (Fig. 4). Assembly is reestablished following tillage, but colonization is influenced by localized abundance of potential colonizers and the assemblage of organisms remaining which can exert an influence upon potential immigrating species—termed priority effects (53). Despite this increased stochasticity, prokaryote phylotype assemblages in arable and bare fallow soils are distinct, both from grassland and each other (Fig. 5 and 6 and Fig. S7). This suggests several possible phylotype assemblages, dependent upon priority effects and the degree of disturbance, even under the same environmental conditions and species pool. Given the regular disturbance, it is unlikely that the phylotype assemblages represent stable endpoints; it is more likely that they reflect alternative transient states (54). Unlike microbial communities in Colorado soils subject to wildfire disturbance, communities of these tilled soils are subject to stochastic assembly for long time periods. Phylotype assemblages are dependent upon disturbance periodicity and may never reach stable endpoints (54). Confirmation of this would require multiyear observation of the communities which was beyond the scope of this study. Although we have not tested it, observation of a greater role for stochasticity in phylotype assembly in disturbed soils suggests that they may be more susceptible to immigration of pathogens, a potential problem in arable soils. Phylotype assemblages may contribute to the significantly reduced yields observed when wheat is grown in the bare fallow soil studied here (22). For fungal assemblages, there was no evidence of increased dispersion in response to tillage. Instead, phylogenetic dispersion was reduced significantly in arable soil compared to grassland (Fig. 4). This suggests strong environmental filtering of phylotypes (niche assembly). This filtering of fungal phylotypes cannot be due to tillage, since phylogenetic dispersion of fungal assemblages in bare fallow soils was equivalent to grassland. Instead inorganic fertilization, or more likely, the fungicide prothioconazole {2-[2-(1-chlorocyclopropyl)-3-(2-chlorophenyl)-2-hydroxypropyl]-1*H*-1,2,4-triazole-3-thione} added as a wheat seed coat is likely to exert a significant selection pressure on fungi in arable soils, resulting in the observed increase in fungal niche assembly.

In summary, after a minimum of 52 years of continuous management, soils experiencing combinations of chemical and physical press and pulse disturbances harbored distinctly different microbial communities with altered community-aggregated traits than undisturbed grassland soil. The effects of each imposed management upon SSU rRNA gene phylotype diversity were kingdom dependent. The observations were also dependent upon whether diversity metrics considered SSU rRNA gene phylogenies. As an example, grassland bacterial phylotype distribution was highly uneven, and the soils were associated with the fewest number of dominant phylotypes which were however more phylogenetically diverse than the greater number of dominant phylotypes in disturbed arable and bare fallow soils. At the other extreme, grassland had the greatest number of dominant fungal phylotypes, but these phylotypes were associated with reduced phylogenetic diversity compared to arable and bare fallow soils. We also observed a distinct influence of different disturbance types upon the assembly of communities. Physical disturbance by tillage increased the influence of stochastic process upon assembly, leading to apparently stable transient states of the prokaryotic communities. Fungal community assembly was not influenced by physical disturbance but showed a strong influence of niche assembly probably due to fungicide incorporation in arable soils.

## MATERIALS AND METHODS

### Soils.

We analyzed soil from plots of the Rothamsted Highfield Ley-Arable field experiment (00:21:48°W, 51:48:18°N). The soil is a silty clay loam (25% clay, 62% silt, 13% sand) (Chromic Luvisol according to FAO criteria). We sampled plots which had been managed consistently as bare fallow for 52 years, arable for 62 years (continuous winter wheat, Triticum aestivum L., at the time of sampling cv. “Hereward” seed treated with Redigo Deter, a combination fungicide-insecticide [Bayer Crop Science]) or mixed grass swards since at least 1838. Grassland and arable plots were established as 300 m^2^ plots, randomly distributed between four in-field blocks. Arable plots receive ammonium nitrate fertilization to provide approximately 220 kg of N ha^−1^ annum^−1^ and an additional 250 kg of K ha^−1^ and 65 kg of P ha^−1^ every 3 years. Bare fallow plots were added later in 1959.

### DNA extraction and metagenome sequencing.

Soil was collected from triplicate plots for each treatment to a depth of 10 cm using a 3-cm diameter corer. The top 2 cm of soil containing root mats and other plant detritus was discarded. Ten cores per plot were pooled and thoroughly mixed while sieving through a 2-mm mesh; samples were then frozen at −80°C. All implements were cleaned with 70% ethanol (vol/vol) between sampling/sieving soil from each plot. Soil community DNA was extracted from a minimum of 2 g soil using the MoBio PowerSoil DNA isolation kit (Mo Bio Laboratories, Inc., Carlsbad, CA) with three replicates for each soil treatment. When necessary, extracts from individual extractions were pooled to provide enough material for sequencing for each replicate. Ten micrograms of high-quality DNA was provided for sequencing for each of the nine plots. Shotgun metagenomic sequencing of DNA was provided by Illumina (Great Abington, UK) using a HiSeq 2000 sequencing platform, generating 150-base, paired-end reads. The generated sequences were limited to a minimum quality score of 25 and a minimum read length of 70 bases using Trimmomatic (55). After filtering to remove substandard sequences, the average metagenome sizes for each soil were 4.96 × 10^8^ reads for grassland, 2.86 × 10^8^ for arable soil, and 2.88 × 10^8^ for bare fallow soil.

### Estimation of community-aggregated traits.

We selected two community-aggregated traits (CATs) to test our hypothesis regarding the opportunity space provided by the treatments studied. First, we generated information regarding the average genome length (AGL) of prokaryotes in each soil metagenome using the ags.sh binary (28). The process proceeds in several steps. First, the abundance of a set of 35 single-copy genes were enumerated, and coverage was estimated as the total number of annotated bases divided by each gene length. These largely translation-associated marker genes occur only very occasionally as duplicates within genomes, are considered both essential for cellular life and very ancient, evolve at a low rate, and code for basal cellular processes, exhibiting little variation across phyla (29). The number of distinct genomes present in each metagenome was then calculated as the average coverage of the 35 single-copy genes. AGL was derived from the ratio of the total number of bases in a data set to the number of genomes identified in the data set. Second, we calculated the average copy number of the 16S rRNA gene using the acn.sh binary (28) which estimated the 16S rRNA gene coverage as the ratio of bases annotated as belonging to the 16S rRNA gene using SortMeRNA version 2.0 (56) and the 16S rRNA gene length (1,542 bases from Escherichia coli). This value was then divided by the number of genomes in the metagenome described above to estimate the average copy number.

### SSU rRNA gene phylogenetic placement.

Each of the metagenomes generated in this study was analyzed to assess the phylogenetic diversity of bacterial, archaeal, and fungal SSU rRNA genes. Nucleotide-based profile hidden Markov models (pHMMs) were generated from multisequence alignments (MSAs) of reference sequences of each gene using HMMBUILD, part of the HMMER suite version 3.1 (57). All MSAs were generated using the 1PAM/κ = 2 scoring matrix and the *E-INS-i* iterative refinement algorithm in MAFFT version 7.3 (58). For 16S rRNA genes, pHMMs were generated from alignment of a set of 7,245 bacterial and 266 archaeal curated reference sequences associated with PAPRICA version 0.5.2 (59), built November 2019. For the fungal 18S rRNA gene, a pHMM was generated from 2,447 reference sequences downloaded from the National Center for Biotechnology Information’s curated Fungal 18S Ribosomal RNA RefSeq Targeted Loci Project, built February 2020. Metagenome reads with homology to each pHMM were identified using HMMSEARCH and a 1 × 10^−5^ expect-value (*E*) cutoff. Each homologous read was assigned to branches of maximum likelihood (ML) phylograms generated from the respective reference gene sets using RAxML version 8.2.4 (60). Phylogenetic placement of exact sequence variants was implemented using EPA-NG version 0.3.6 (61) and visualized using iTOL version 5.5 (62). Gene sequence placements can be translated into robust relative abundance and phylogenetic relatedness estimates of organisms using the taxonomic labeling of phylogram branches.

### Statistical analyses.

To test our hypotheses, we generated several gene assemblage-related metrics, including gene sequence richness and phylogenetic diversity, abundance-sensitive measures of sequence and phylogenetic diversity using a one-parameter family of diversity measures, balance-weighted phylogenetic diversity (*BWPD*_θ_) (63) and phylogeny-based distance metrics for assemblage comparison between treatments. Sample size- and coverage-based interpolation and extrapolation of *^q^D* of each SSU rRNA gene was performed using iNEXT version 2.0.20 (64) in R version 3.6.1, treating each read as a point mass concentrated on the highest-weight placement. Extrapolation of *^q^D* was extended to the greater of the maximum number of sequences across all samples or twice the number of sequences in the smallest sample; 77,805 bacterial 16S rRNA sequences, 62,304 archaeal 16S rRNA sequences, and 15,153 fungal 18S rRNA sequences. Estimates of associated 95% confidence intervals were based on 399 bootstrap samples (65).

Estimates of gene sequence similarity-sensitive phylogenetic diversity (PD) based upon placement of homologous metagenomic reads were assessed by computing a measure incorporating abundance, using the FPD binary in GUPPY version 1.1 (part of the PPLACER code [66]), accounting for reference ML phylogram pendant branch length. The effects of different land managements upon *BWPD*_0_ and *BWPD*_1_ were analyzed using one-factor analysis of variance (ANOVA) after testing for homogeneity of variances using Levene’s test and normality using the Shapiro-Wilk test. We calculated omega squared (ω^2^) as an estimate of the extent to which variance in the response variable was accounted for by the treatment (effect size). The experimental design was limited by having only three replicate plots per land management and as a result low statistical power (increasing the likelihood of type II error). Where significant treatment effects were identified, *post hoc* pairwise comparisons were performed using the Tukey-Kramer Studentized *Q* statistic, following the Copenhaver-Holland procedure of sequentially rejective multiple comparisons (67) to control family-wise type I error. All univariate tests were performed using PAST version 4.02 (68). An α of 0.05 was considered significant.

To assess prokaryotic 16S rRNA and fungal 18S rRNA gene-based β-diversity between land managements, Kantorovich-Rubinstein (KR) metrics of phylogenetic distance were calculated from phylogenetic placements of metagenome reads using the KRD binary associated with GAPPA version 0.4.0 (69), treating each query as a point mass concentrated on the highest-weight placement. The KR distance metric, which is allied to the weighted-UniFrac measure (70), compares gene assemblage distributions on a phylogram in units of nucleotide substitutions per site, a biologically meaningful approach to comparing communities. Comparison of β-diversity dispersion of KR phylogenetic distance metrics within and between land management was performed using a multivariate analogue of Levene’s test for homogeneity of multivariate variances, the PERMDISP test (71). Differences in gene assemblages based upon KR distance metrics were tested using permutational multivariate analysis of variance (PERMANOVA) (72). In addition, the distinctiveness of bacterial, archaeal, and fungal phylogenetic assemblages associated with each land management was tested in multivariate space using canonical analysis of principle coordinates (CAP) (73), maximizing the success of a leave-one-out allocation to land management to determine the appropriate number of axes to include in the test. CAP-based hypothesis testing was based upon the sum of canonical eigenvalues. For all multivariate tests, probability estimation was based upon 99,999 permutations (denoted as *p*_perm_). Where PERMANOVA indicated a significant treatment effect, pairwise comparisons were performed. However, since the number of observations was insufficient to allow a reasonable number of permutations, Monte Carlo probabilities (denoted *p*_MC_) were calculated based upon an asymptotic permutation distribution. Multivariate tests were performed using PRIMER PERMANOVA+ version 7.0.13 (PRIMER-e, Auckland, New Zealand).

Unconstrained ordination based upon principal-component analysis of the difference in placement masses across reference phylograms—termed edge-PCA (74)—was used for graphical representation of phylogeny-based differences between treatments in a two-dimensional plane using the EDGEPCA binary in GAPPA, treating each query as a point mass concentrated on the highest-weight placement. An advantage of edge-PCA is that branches associated with placements contributing to eigenvalues on each axis, and thus organisms contributing to the observed differences, can be identified. For fungal taxa identified by edge-PCA to be characteristic of the difference land managements, we used the FUNGuild version 1.1 annotation tool (75) to associate taxa with ecological guilds.

### Data availability.

Sequence data associated with this research have been deposited in the European Nucleotide Archive with accession number PRJEB43407. Extensive chemical, climate, and treatment data and history are available on the e-RA database (http://www.era.rothamsted.ac.uk/), maintained by Rothamsted Research.
