# Supplementary figures and images for: Microbiome Aggregated Traits and Assembly Are More Sensitive to Soil Management than Diversity
Source: mSystems. 2021 May 27;6(3):e01056-20. doi: 10.1128/mSystems.01056-20 (PMC8269249; doi:10.1128/mSystems.01056-20)

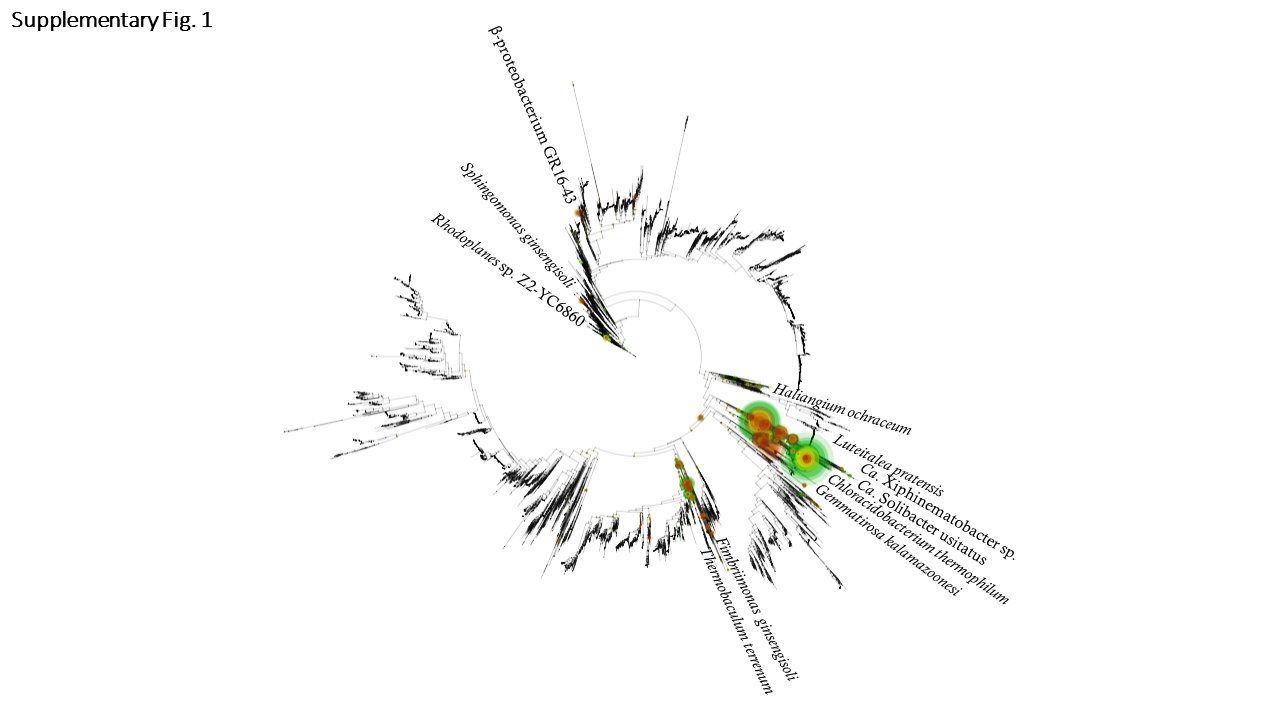

Supplement: FIG S1 [file msystems.01056-20-sf001.tif]

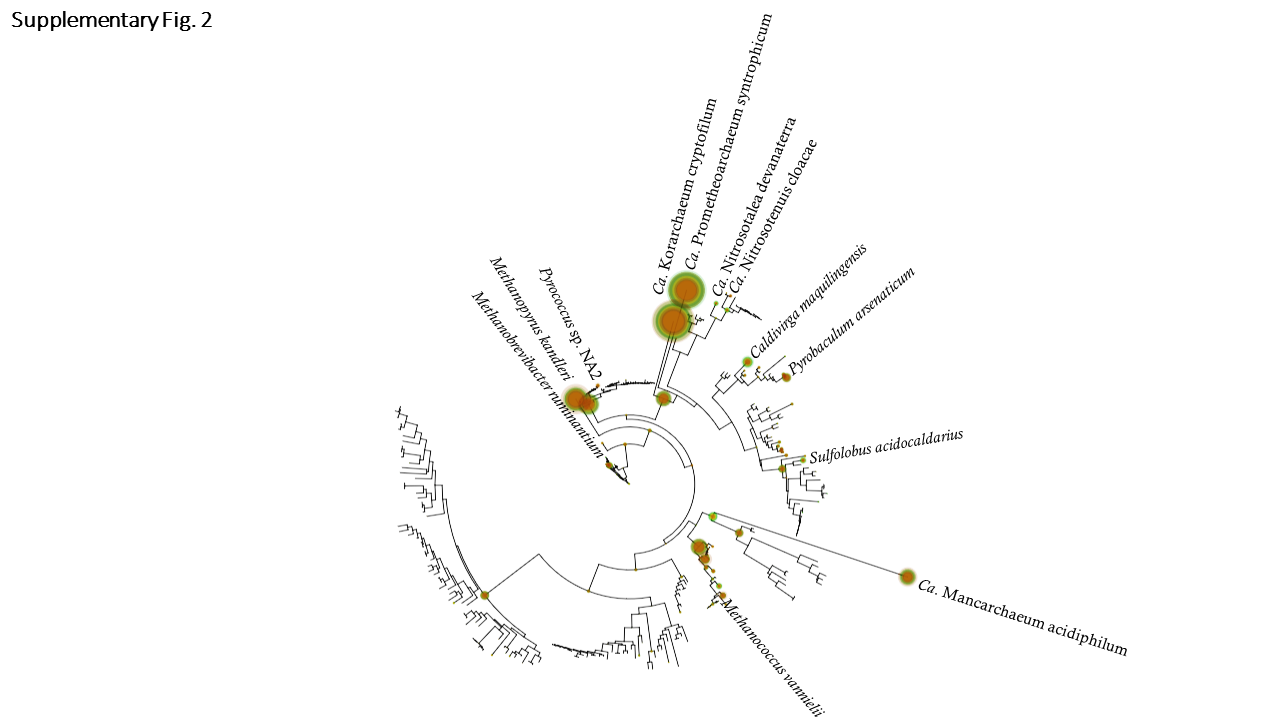

Supplement: FIG S2 [file msystems.01056-20-sf002.tif]

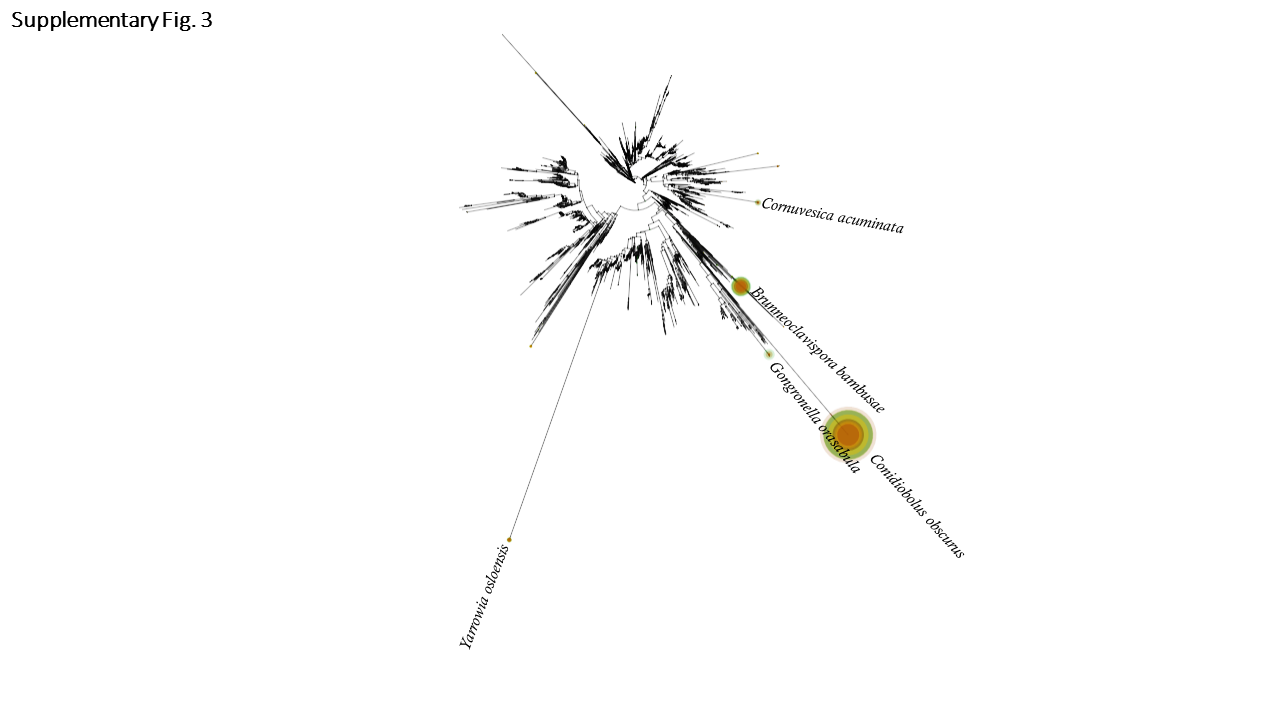

Supplement: FIG S3 [file msystems.01056-20-sf003.tif]

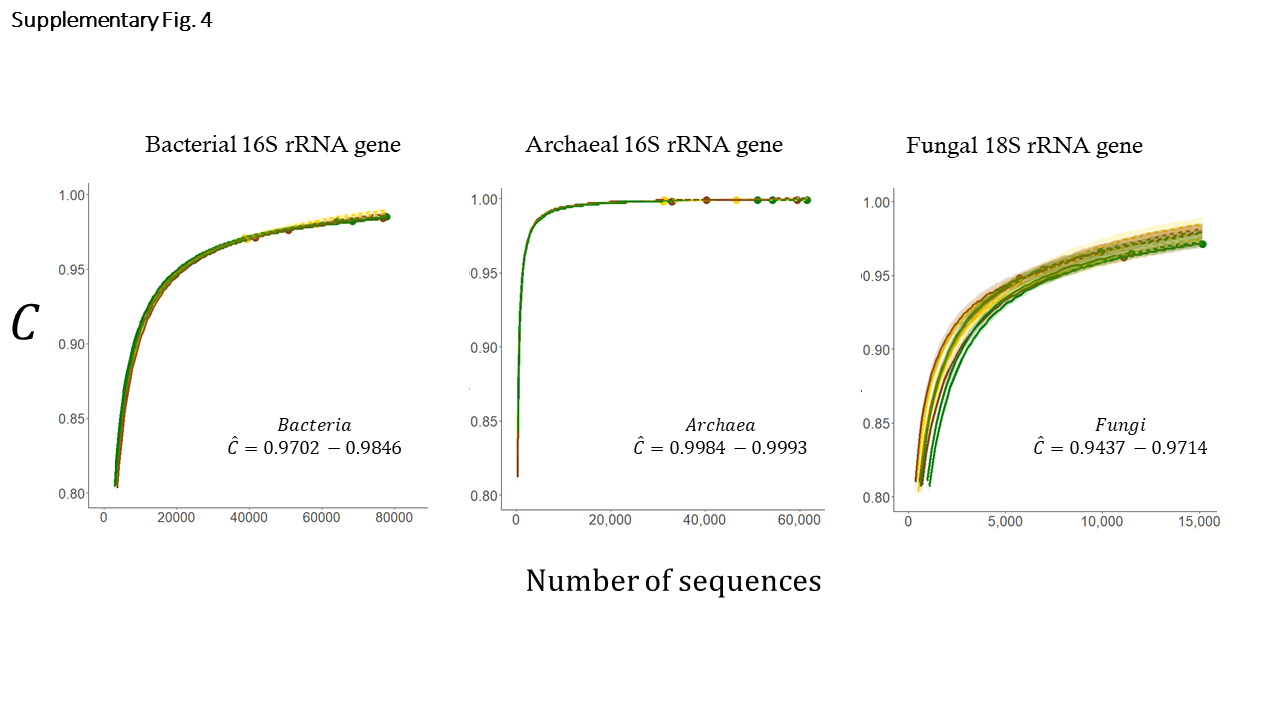

Supplement: FIG S4 [file msystems.01056-20-sf004.tif]

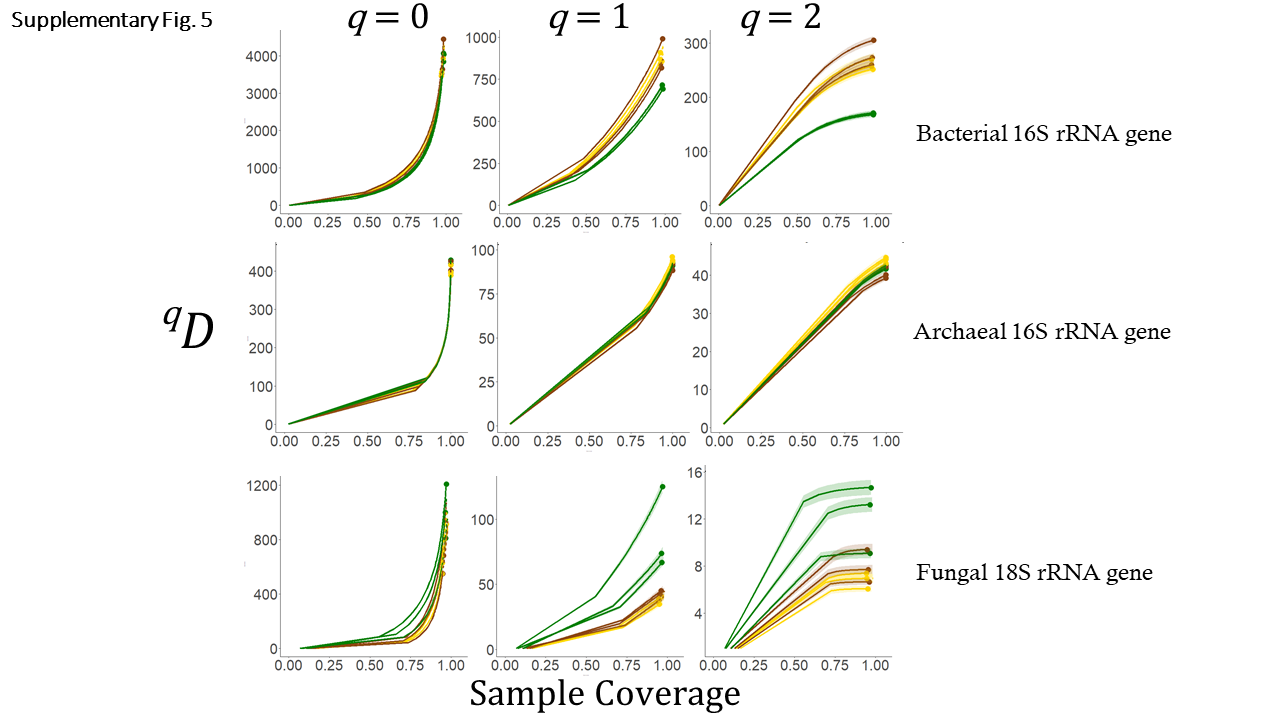

Supplement: FIG S5 [file msystems.01056-20-sf005.tif]

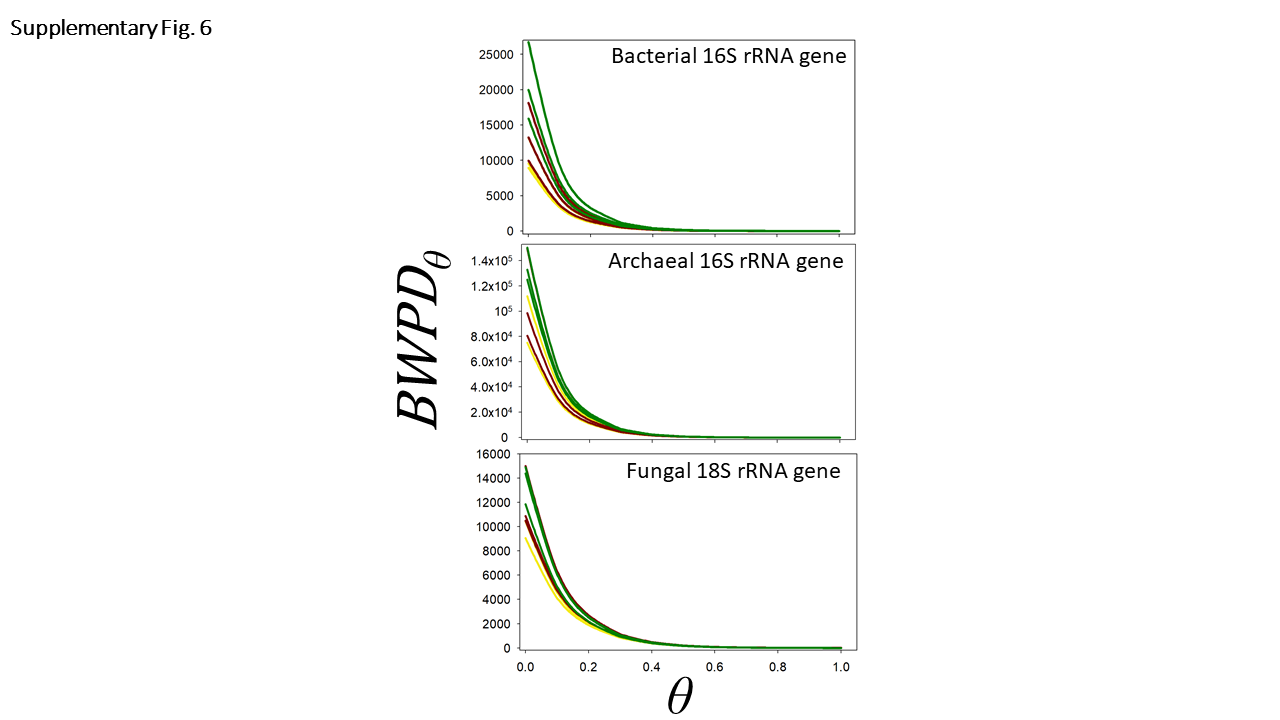

Supplement: FIG S6 [file msystems.01056-20-sf006.tif]

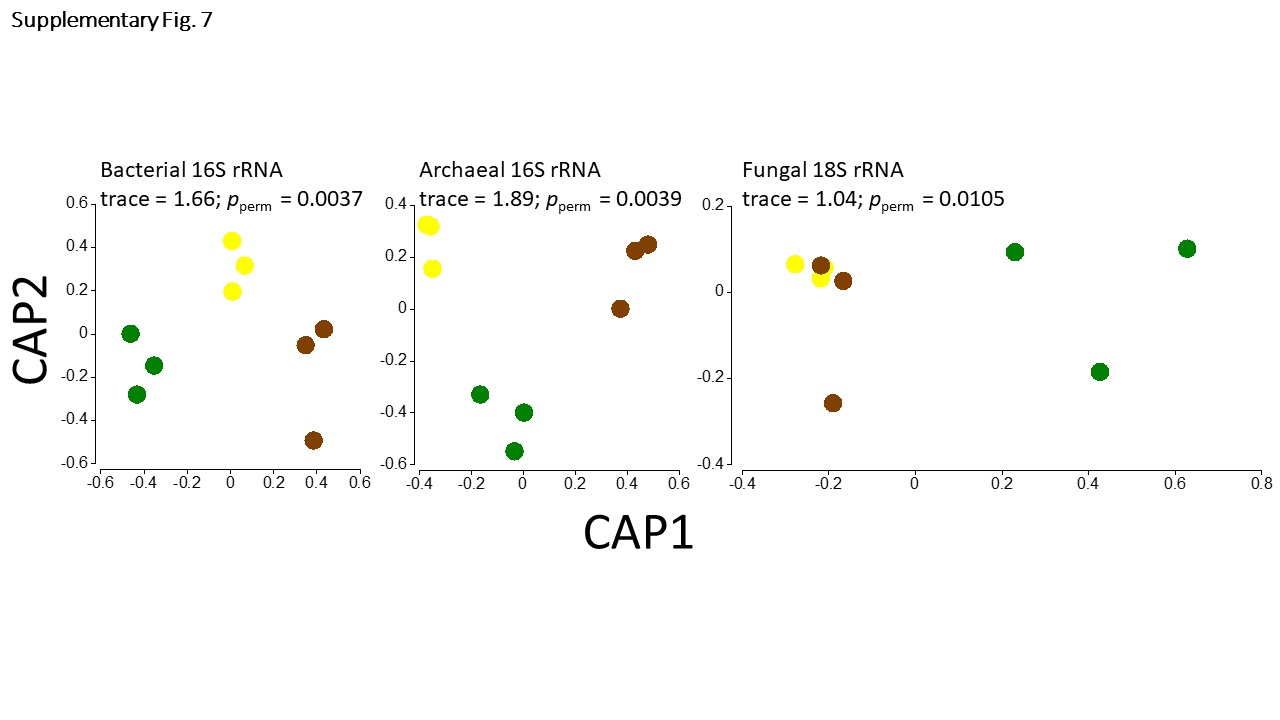

Supplement: FIG S7 [file msystems.01056-20-sf007.tif]
